# Weight Bearing Activities change the Pivot Position after Total Knee Arthroplasty

**DOI:** 10.1038/s41598-019-45694-y

**Published:** 2019-06-24

**Authors:** Philippe Moewis, Hagen Hommel, Adam Trepczynski, Leonie Krahl, Philipp von Roth, Georg N. Duda

**Affiliations:** 1 Julius Wolff Institute, Charité - Universitätsmedizin Berlin, Berlin, Germany; 2 Krankenhaus Märkisch-Oderland GmBH, Wriezen, Germany; 3 Medizinischen Hochschule Brandenburg Theodor Fontane, Neuruppin, Germany; 4 Sporthopaedicum Regensburg, Regensburg, Germany

**Keywords:** Medical research, Engineering

## Abstract

The knee joint center of rotation is altered in the absence of the anterior cruciate ligament, which leads to substantially higher variance in kinematic patterns. To overcome this, total knee arthroplasty (TKA) designs with a high congruency in the lateral compartment have been proposed. The purpose of this study was to analyze the influence of a lateral pivot TKA-design on *in-vivo* knee joint kinematics. Tibiofemoral motion was retrospectively addressed in 10 patients during unloaded flexion-extension and loaded lunge using single plane fluoroscopy. During the unloaded flexion-extension movement, the lateral condyle remained almost stationary with little rollback at maximum flexion. The medial condyle exhibited anterior translation during the whole flexion cycle. During the loaded lunge movement, a higher degree of rollback compared to the unloaded activity was observed on the lateral condyle, whereas the medial condyle remained almost stationary. The results showed a clear lateral pivot during the unloaded activity, reflective of the implant’s geometric characteristics, and a change to a medial pivot and a higher lateral rollback during the weight-bearing conditions, revealing the impact of load and muscle force. It remains unclear if the kinematics with a lateral TKA design could be considered as physiological, due to the limited knowledge available on native knee joint kinematics.

## Introduction

Though the survival rate in total knee arthroplasties (TKA) has improved^1^, a relevant number of patients (20%) remain unsatisfied with the outcome, regardless of specific TKA design features. Across most designs, postoperative knee pain persists without a distinct radiological or clinical reason^2,3^. One of the main known causes is non-physiological kinematics^4^. However, it remains under debate until what extent the surgical technique, such as soft-tissue balancing, and implant geometry, such as curved radii, have a direct influence on the knee joint kinematics^5^.

Despite various developments in TKA implant designs, which include fixed bearing, rotating platform, cruciate retaining and posterior stabilized geometries, it remains unclear which design is superior for achieving a physiological kinematics^6–8^. In particular, studies on the kinematics of symmetrical femoral component designs showed inconsistent femoral anterior-posterior (AP) translation during flexion, which also often report increased patellofemoral and anterior knee pain^9–12^. Furthermore, it has been suggested that such excessive AP motion could result in bony impingement between the femur and the posterior rim of the tibial insert^13–16^.

If a proper TKA component alignment as well as ligament balancing has been conducted, a proper soft tissue guidance of the knee replacement may be retained^16–19^. However, excessive ligament tensioning limits the extent of movement, while loose ligaments leads to knee joint instability^20,21^, both of which can be reasons for revision surgery^22–24^. Both instability and unbalanced ligament structures may lead to pronounced asymmetric polyethylene (PE) wear and thus, increased loosening rates^24^.

It has been reported that the native knee requires a specific degree of rollback of the lateral compartment coupled with a medial pivot^25^, which leads to an external axial rotation of the femur. This rollback is thought to be even more pronounced with increasing knee flexion angles to enable deep flexion without excessive shear forces acting at the patella or overloading the extensor mechanism^25^.

On the other hand, previous reports on the kinematics of healthy knees have shown a predominantly lateral pivot during low flexion activities such as walking and running^26–28^. In the absence of the anterior cruciate ligament (ACL), the center of rotation is altered from a medial to a lateral pivot, as reported by Yamaguchi and Isberg’s analysis of squats performed in ACL-deficient subjects^29,30^. In a similar analysis using fluoroscopy, Dennis and colleagues also reported an altered pivot as well as substantially higher variance in the pattern and magnitude of both AP translation and axial rotation^31^. In response, designs with high congruency in the lateral compartment have been developed and implemented in an attempt to overcome this. Such highly congruent lateral femoral condyle designs are consequently combined with a widened medial condyle to increase contact area and to minimize contact pressures^32^. Designs with this specific characteristics are named lateral pivoting designs.

Previous *in vitro* analyses of lateral pivoting designs have shown a change of the pivot position at 10 and 60 degrees of knee flexion^33^. Different reports on *in vivo* static analysis during kneeling and lunge activities have shown either a predominantly lateral pivot^34,35^ or a lateral rollback^16,36^. However, a comprehensive analysis of knee kinematics under weight-bearing or non-weight-bearing dynamic conditions in a set of TKA patients, particularly for demanding knee flexion activities, is currently lacking in the literature.

The purpose of this study was to analyze the influence of a lateral pivot TKA design on *in vivo* knee joint kinematics during knee flexion activities and to compare this to corresponding clinical data. Patients were analyzed at 2 years post-surgery to allow for a postoperative status that is not influenced by the current stage of rehabilitation. We hypothesized that kinematics in a TKA implant designed to exhibit a lateral pivot during the range of knee joint flexion would differ between loaded and unloaded knee flexion activities.

## Materials and Methods

### Patients

In a retrospective study, 10 subjects (66.1 years mean age (SD 6.1), 6 females, 33.2 mean BMI (SD 5.0)) were included for this analysis. All subjects were implanted with the 3D Knee™ fixed bearing cruciate retaining TKA design (DJO GLOBAL), which has an asymmetric femoral component design. This TKA exhibits high lateral congruency in extension, which decreases at higher flexion, as well as a widened medial condyle. Inclusion criteria were a primary diagnosis of osteoarthritis with a coronal deformity of <10°, as well as no previous open knee surgery. Patients were measured at 24 months post-surgery. The study was approved by the local ethics committee (Landesärztekammer Brandenburg, Germany, approval-Nr: S9 (A)/2016). All subjects provided written informed consent prior to participation. All investigations were performed in accordance with relevant guidelines/regulations.

### Surgical technique

All surgeries were conducted under full general anesthesia, with standard pain management through blockades of the femoralis and ischiadicus nerves. A measured resection technique was applied and a constant tibia slope of 5° was maintained. The posterior cruciate ligament (PCL) was preserved and balanced with a spacer technique. All implants were fully cemented with reference to manufacturer guidelines. All surgeries were performed by the same surgeon, who was neither involved in data acquisition, data interpretation, nor preparation of the manuscript. Standard post-operative standard physiotherapy, pain treatment and clinical monitoring were conducted.

### *In vivo* 3-D knee kinematics using single plane fluoroscopy

Single plane fluoroscopy analysis was conducted in all subjects using a Philips BV Pulsera device (Philips Medical Systems GmbH, Hamburg, Germany) to assess the tibiofemoral kinematics (30 Hz frame rate, 8 ms pulse width, beam energy 60 kVp, beam current 5 mA). Image resolution was 1024 × 1024 pixels with a 12-bit color depth. Additional images of a specially designed Perspex calibration box^37^ were collected to correct for image distortion.

All patients performed a single leg weight-bearing activity (lunge) and a single leg unloaded activity (flexion-extension) on the TKA limb to assess tibiofemoral knee kinematics under closed and open chain conditions, respectively. Special care was taken to clarify the activities to the subjects and to ensure correct performance during data collection.

**Figure 1:**
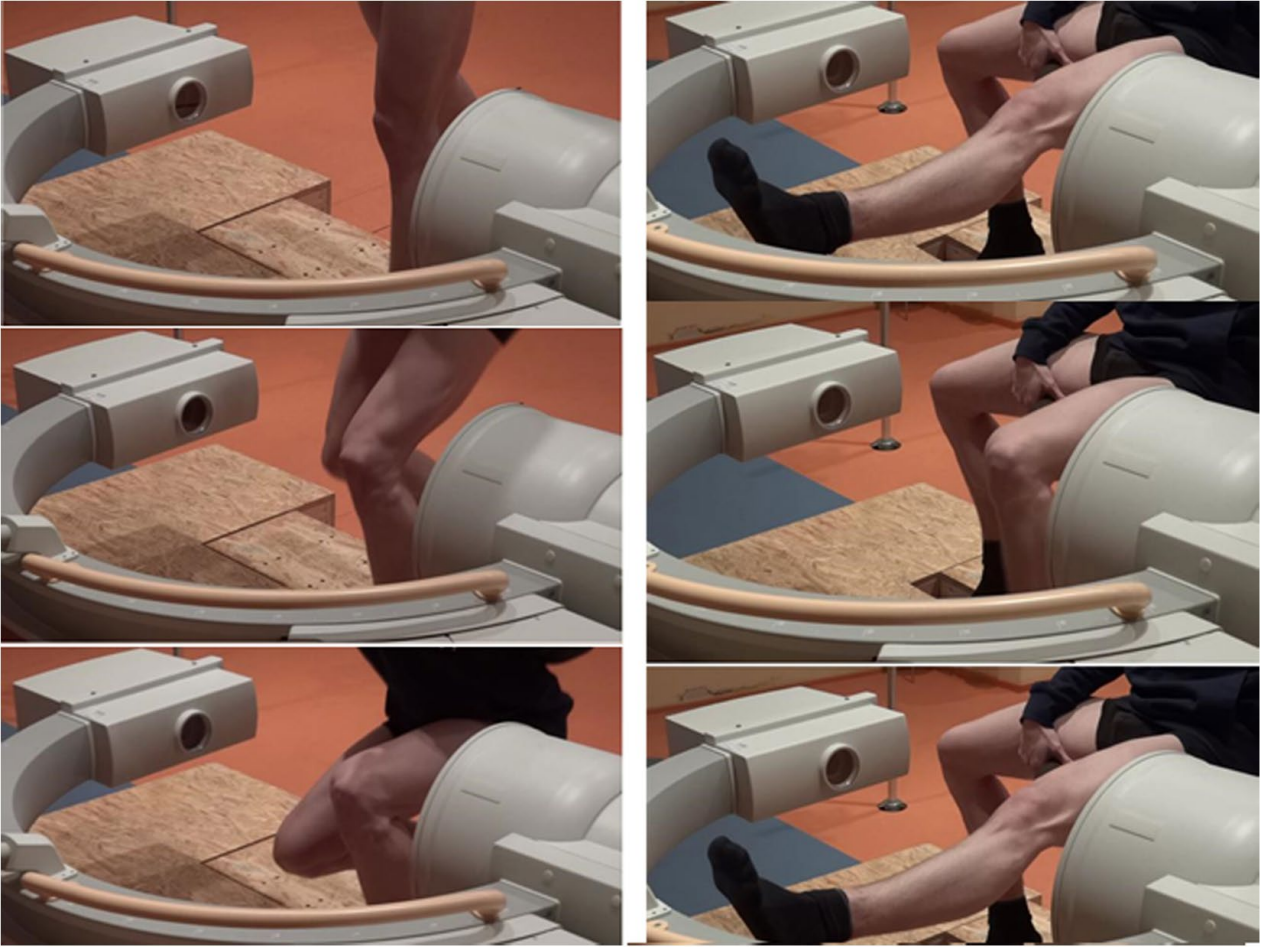
Left: Weight-bearing lunge. Right: Unloaded flexion-extension.

During the lunge, both feet were at the same level but with the foot of the leg of interest positioned frontally to achieve a position of the knee to be analyzed as near as possible to the center of the image intensifier. The contralateral leg was in a more posterior position to avoid overlapping. The activity started at full extension and was conducted without a break until the maximal active possible knee flexion was achieved without help and was completed when full extension was reached again (Fig. 1).

The unloaded flexion-extension activity started also at full extension and was conducted without a break until maximal active possible flexion and finalize at full extension (Fig. 1). Three repetitions were collected during both activities. Considering the frame rate of 30 Hz and the varied duration of the activity (approximately 8–15 seconds), between 250 and 450 frames were collected during each repetition.

### Clinical questionnaires

The following clinical questionnaires were collected: the Knee Society Score (KSS)^38^, consisting of the Knee Score (KS) and Function Score (FS)^38^, as well as Forgotten Joint Score (FJS)^39^, the High Flexion Knee Score (HFKS)^40^, and subjective patient postoperative satisfaction (10 points meaning maximal satisfaction with the prosthesis).

### Data post-processing and analyses

The collected “Digital Imaging and Communications in Medicine” (DICOM) packages of every activity and repetitions were separated into single images. The images starting from maximal extension to maximal flexion were then used for fluoroscopic analysis. The 3D computer-aided-design (CAD) models of the femoral and tibial metallic components were registered to the fluoroscopic images as previously described^41^. Since single plane fluoroscopy is not a direct measurement method, the calculated joint kinematics depend highly on the accuracy of the CAD models^42^ and contour selection. In addition to the automatic contour detection method applied, manual corrections need to be conducted to select the relevant and also to discard the erroneous contour parts. The accuracy of the registration procedure has been analyzed previously under dynamic conditions with root mean square values of 0.2–0.6 mm for translations and 0.4–0.8 for rotations reported^41^.

**Figure 2:**
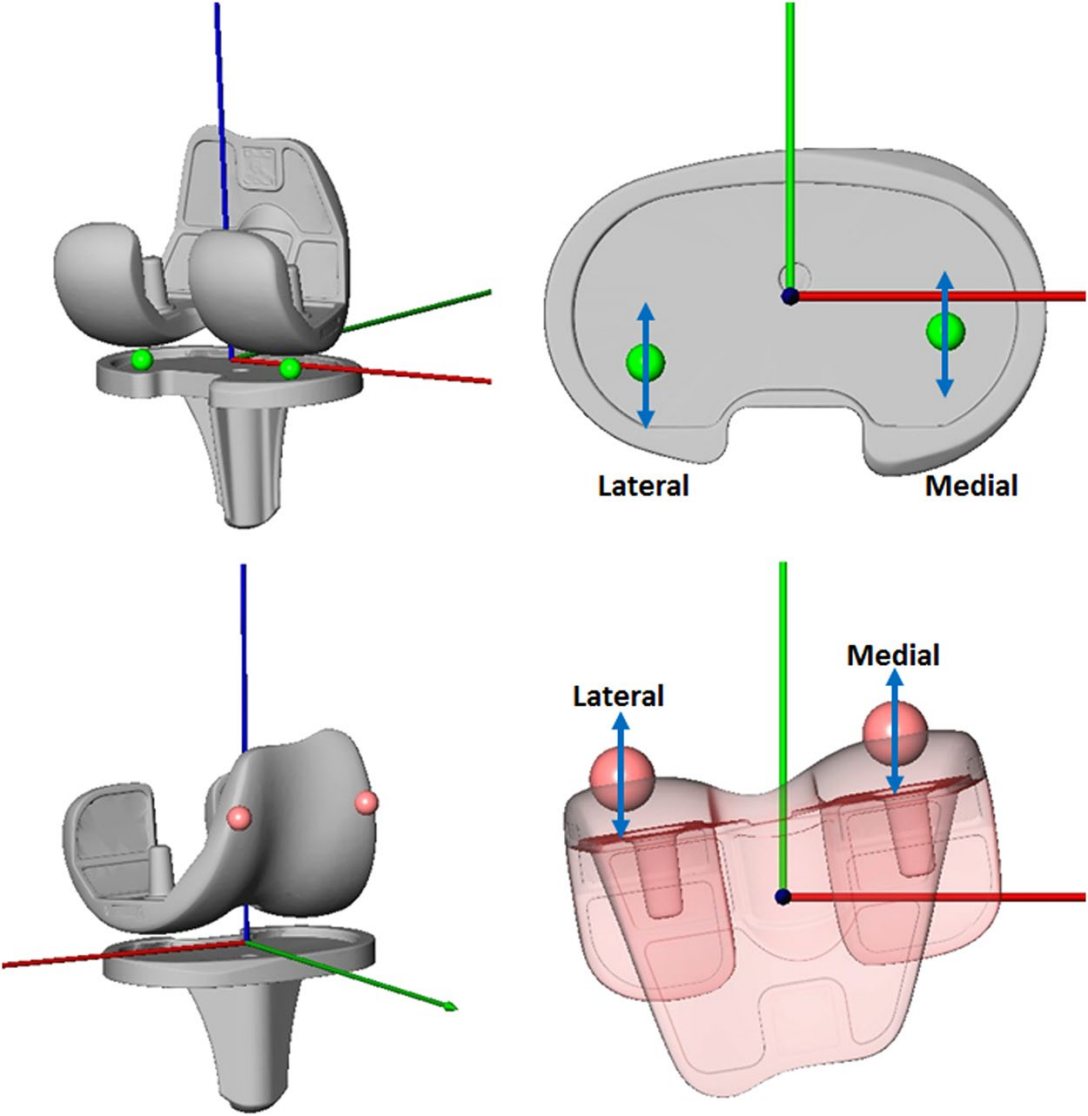
3-D Surface models of the total joint replacement implant. Upper left: distal lateral and medial condyle points (shortest distance between femur and tibia). Bottom left: most anterior tangency lateral and medial condyle points (shortest distance between femur and anterior frontal plane). Upper/bottom right, determination of the anterior-posterior translation with the medial/lateral distal and medial/lateral anterior tangency points respectively.

The transformations after the registration procedure were used to reproduce the positions and orientations of the components using the AMIRA environment (Visage Imaging, Berlin, Germany). The origin of the tibial coordinate system was defined as the intersection of the tibia plateau with the tibia shaft axis, which also formed the Z-axis (pointing proximally), while the X-axis pointed right and the Y-axis anterior. The most distal points of the medial and lateral femoral condyles were determined and projected onto the tibia component plateau to generate a distal line^43^ (Fig. 2).

The axial rotation was defined as the angle between the distal line and the medio-lateral tibial axis. A frontal plane, perpendicular to the tibia plateau plane and positioned anteriorly, was used to determine the most anterior tangency medial and lateral femoral frontal points as a reference for the movement of the patella^43^ (Fig. 2). Both AP translations were expressed as the absolute values of the distal/anterior points relative to the origin of the tibial coordinate system. These values were resampled at 1° increments of knee flexion using linear interpolation to allow for determination of cohort means and standard deviations.

To analyze the relationship between kinematics and clinical outcomes, a correlation analysis was conducted between the FJS values and the AP translation at maximal achieved knee joint flexion for both the medial and lateral compartments.

## Results

A clear variability in the initial position of the lateral and medial distal points can be observed in both activities in the extended position (Appendix Fig. 1).

During the analysis of the flexion-extension activity, 7 subjects showed a relatively stationary position of the lateral condyle with additional little rollback at maximum flexion. On the other hand, the medial condyle continuously exhibited anterior translation during the whole flexion cycle in all patients (Appendix Fig. 1). In general, the subjects attained a high maximum flexion degree during this active non weight-bearing activity, with a flexion of 89.1 +/− 8.3° (mean +/−SD).

During the weight-bearing lunge, the lateral condyle showed a higher extent of rollback, which started approximately at mid-flexion, while the medial condyle remained stationary with additional anterior translation at approximately 50–60 degrees of flexion, although in less magnitude compared to the flexion-extension activity, (Appendix Fig. 1). Compared to the unloaded activity, less knee joint flexion could be reached by the subjects, with a flexion of 65.5 +/− 18.0°. The mean and standard deviations of the absolute values of this analysis are displayed in Table 1.

**Table 1:** Mean and standard deviations of the absolute AP kinematics of the distal and anterior tangency points during flexion-extension (flex-ext) and lunge activities, negative values indicate a posterior translation.

		Distal Points				Anterior Tangency Points			
		Flex-Ext (mm)		Lunge (mm)		Flex-Ext (mm)		Lunge (mm)	
		Medial	Lateral	Medial	Lateral	Medial	Lateral	Medial	Lateral
Implant Flexion Angle (°)	0	−4.6 ± 1.4	−8.9 ± 1.1	−4.4 ± 2.4	−9.2 ± 1.7	28.1 ± 3.0	24.5 ± 2.7	28.5 ± 3.0	24.1 ± 2.1
	10	−4.7 ± 1.5	−9.2 ± 1.3	−4.1 ± 1.6	−9.7 ± 2.2	27.5 ± 3.0	23.6 ± 2.7	28.0 ± 2.7	22.9 ± 2.2
	20	−4.3 ± 1.7	−9.2 ± 1.6	−3.6 ± 1.1	−10.1 ± 2.7	27.3 ± 3.1	22.8 ± 2.8	27.6 ± 2.3	21.6 ± 2.5
	30	−3.7 ± 2.0	−9.1 ± 1.7	−3.3 ± 1.2	−10.5 ± 3.1	27.3 ± 3.2	22.2 ± 2.7	27.4 ± 2.1	20.5 ± 2.9
	40	−3.2 ± 2.3	−9.1 ± 1.8	−3.0 ± 1.3	−10.9 ± 3.4	27.5 ± 1.2	21.7 ± 2.6	27.3 ± 1.9	19.6 ± 3.1
	50	−2.4 ± 2.6	−9.2 ± 1.9	−3.0 ± 1.2	−11.5 ± 3.7	27.9 ± 3.4	21.2 ± 2.5	27.1 ± 1.8	18.6 ± 3.6
	60	−1.5 ± 2.6	−9.5 ± 1.8			28.5 ± 3.2	20.6 ± 2.3		
	70	−0.8 ± 2.6	−9.7 ± 1.8			28.8 ± 3.1	19.7 ± 2.3		
	80	0.4 ± 2.6	−10.0 ± 2.4			28.4 ± 3.1	17.5 ± 2.7		

**Figure 3:**
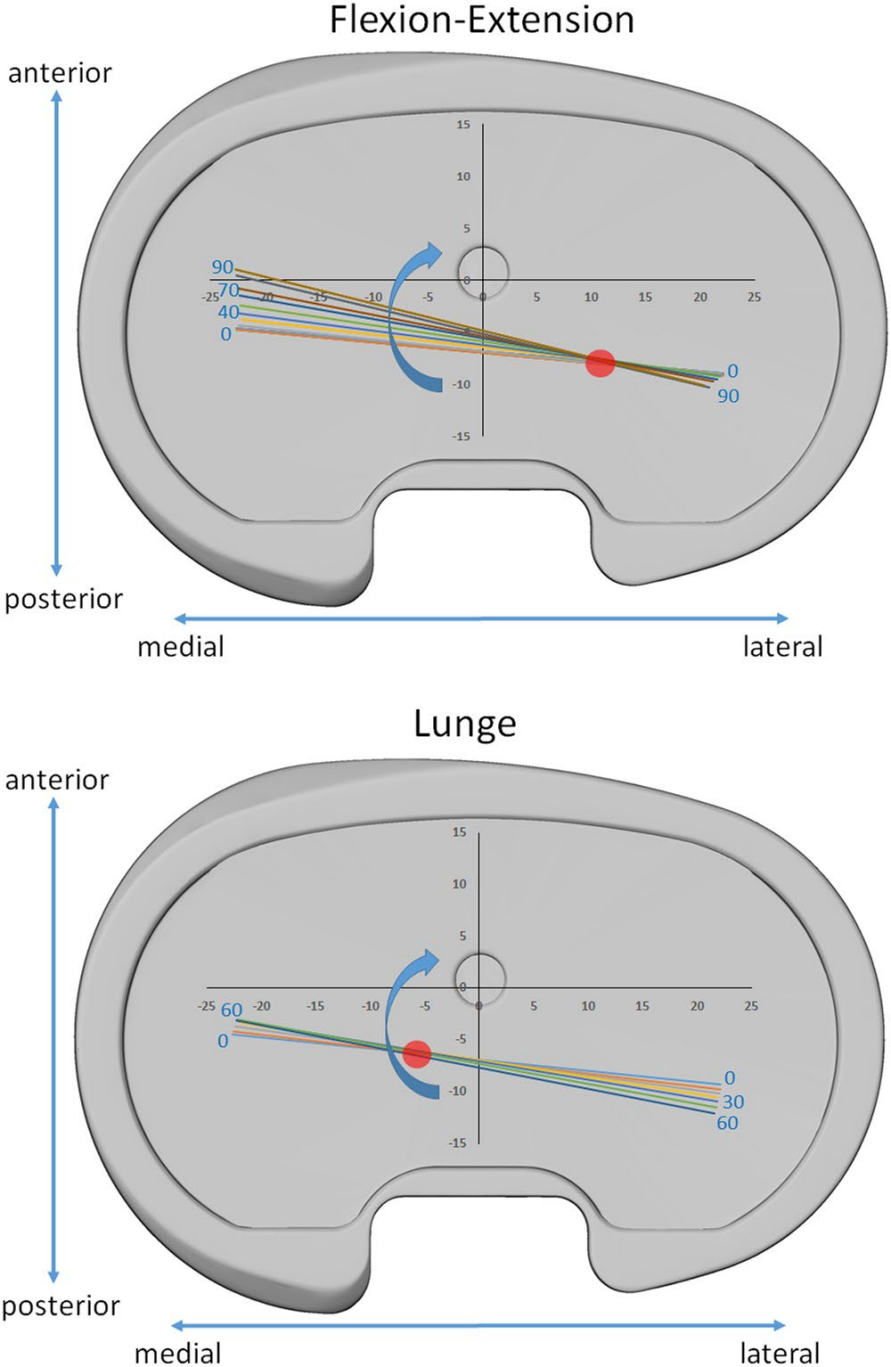
Tibiofemoral kinematics (averaged across all 10 subjects) for both activities. Blue arrow indicates the femoral component rotation, red circle indicates the position of the pivot.

Considering the axial rotation, the initial anterior position of the medial, as well as the posterior position of the lateral distal points indicates an externally rotated position of the femoral component during extension. An increase of this external rotation was then identified in both measured activities with values at maximum flexion of 9.2 +/− 3.1° and 4.8 +/− 3.3° for the flexion extension and lunge respectively. A clear change of pivot from the medial compartment during the flexion-extension activity to the lateral compartment during the lunge activity could be observed (Fig. 3).

The medial and lateral anterior tangency points as reference of the movement towards the patella showed a continuous posterior translation of the lateral point in both activities. On the other hand, the medial point remained relatively stationary at the beginning of the unloaded flexion-extension activity, translating posteriorly at approximately 70° of knee joint flexion. While this posterior translation was relatively constant, although minor compared to the lateral point during lunge (Appendix Fig. 2). The mean and standard deviations of the absolute values of this analysis are displayed in Table 1.

Regarding the clinical questionnaires, a clear good passive flexion was observed in all subjects (mean: 133°, range: 125–145°) as well as a high subject’s satisfaction with the prosthesis (mean: 8.2, range: 5–10). A clear variability could be observed in the values of the FJS and HFKS. This information is collected in Table 2. The correlation analysis showed low correlation values (maximal R^2^ = 0.3478), however a tendency towards a reduced anterior position at high FJS values could be observed (Appendix Fig. 3).

**Table 2:** Postoperative clinical data.

Patient	24 Months							
	Extension (°)	Max. passive Flexion (°)	KS	KS (F)	KSS	FJS	HFKS	Patient Satisfaction (1–10)
1	5	130	78	90	168	71	32	7
2	0	145	100	100	200	94	55	10
3	0	130	57	90	147	21	23	5
5	5	135	87	80	167	71	43	8
6	0	135	90	80	170	62	40	9
7	0	145	95	80	175	78	35	7
9	0	130	90	80	170	68	39	10
10	0	125	84	90	174	38	26	8
12	0	130	87	90	177	70	34	9
13	0	125	83	100	183	57	40	9
Mean		133	85.1	88	173.1	63	36.7	8.2

## Discussion

Tibiofemoral kinematics after TKA are determined by multiple factors such as patient-specific characteristics, surgical technique, soft-tissue balance, PCL strain, limb alignment, bearing design and femoral component geometry^43^. Inconsistent anterior translation of the femur in flexion has been previously observed in symmetrical femoral components, leading to a large variety of implants with their own advantages and limitations^6–8^. The analyzed implant in this study has a congruent lateral compartment with the aim to provide more AP stability and to prevent paradoxical motion in early and mid-flexion. On the other hand, the less conforming medial compartment aims to reduce the contact pressure and to drive femoral rollback^32^. Despite these targeted features, the analysis conducted in the present study shows different behavior during unloaded and loaded activities.

There is a clear anterior translation in the medial compartment during the unloaded flexion-extension accompanied by a relative stationary position, or pivoting, on the lateral compartment. This leads to the assumption that a knee joint kinematics reflective of the implant’s geometric characteristics takes place during this activity. However, during the loaded lunge activity, an anterior shift in the medial compartment is reduced and is accompanied by increased lateral rollback. If patients would have been able to achieve a higher knee joint flexion, this observed movement pattern may have been continued at higher knee flexion angles due to the progressively decreasing of the congruency. The lateral rollback observed is comparable to the reports on the same prosthesis design of previous studies. Posterior positions of the lateral condyle up to 7, 8, 9 and 15 mm have been also reported by Mikashima, Ginsel, Watanabe and Harman respectively^16,34–36^. However, such degrees of rollback were achieved in kneeling activities performed differently.

Importantly, a clear change of pivot was reported between the measured activities: while a clear lateral pivot was observed during the unloaded activities, a change towards a medial pivot was observed in the loaded lunge, likely due to the influence of muscle activation and axial load during closed chain activities^44^. Apart from this changed pivot, a clear external axial rotation of the femoral component relative to the tibia component was evident in both activities; however, the performance was location- and activity-dependent. The medial pivot during the loaded activity was coupled with an external rotation and a rollback of the lateral condyle, similar to the reports of Pinskerova and colleagues on healthy knees from extension to flexion^25^. However, the lateral pivot during the unloaded activity showed an external rotation coupled with an anterior sliding of the medial condyle. In contrast, this motion pattern has been observed in unconstrained TKA designs with up to 20% more prevalence of a lateral pivot during stair navigation^15^. It is also similar to the reports on healthy knees during walking and running^26–28^ and to the anterior translation of around 8 mm observed during knee flexion in the medial condyle of subjects with ACL deficient knees^29^.

The anterior shift of the medial condyle can be considered paradoxical and it has been shown that tibiofemoral roll-forward leads to increased patello-femoral contact forces^4,45,46^. However, the analysis of the anterior tangency points as reference of the movement towards the patella showed a shift towards posterior of the lateral and medial points, starting medially at 70° of knee joint flexion and continuously from extension to maximal flexion on the lateral compartment. This movement pattern could eventually leads to a relief of the patello-femoral contact force.

The clinical scores show the patients’ high satisfaction with the prosthesis and a tendency towards a reduced anterior position with high FJS values. However, the variability in the Forgotten Joint Score and the High Flexion Knee Score could be related to the limited sample size of analyzed patients. Possible causes of this variability could be the lack of controlled ligament tensioning as well as variable instrumentation.

The general variability observed in our clinical and fluoroscopic data could be related to different factors such as the patient’s level of activity, muscle weakness, body weight index, joint line angulation and loading conditions. Since a measured resection technique was applied during surgery and considering the actual knowledge about gap-balancing and controlled ligament tensioning, the clinical and kinematic performance could benefit from such specific approaches^47,48^.

This study is not free of limitations. First, it is a retrospective study design, which may include selection bias. Though patients were recruited from a single center, operated by single surgeon and treated under standardized protocols to provide comparability, bias may have impacted the findings. Second, only one design with the aforementioned geometric characteristics was analyzed within this study. Thus, any conclusion regarding other implant designs cannot be drawn. Third, pre-operative clinical data was not available, which weaken the interpretation of the post-operative outcome scores.

At 2 years post-surgery, the effect of weight bearing during high flexion activities appears to affect knee joint kinematics in this particular TKA implant. During the unloaded activity, the knee had a clear lateral pivot that was reflective of the implant’s geometric characteristics. Yet during the weight-bearing condition, there was a change to a medial pivot and a higher lateral rollback, which reveals the impact of load and muscle force. It remains unclear if the kinematics with a lateral TKA design could be considered as physiological, due to the limited knowledge available on native knee joint kinematics.

## Supplementary information

Supplementary Info

## Data Availability

All relevant data are within the paper. Additional information associated with this article can be found at: https://osf.io/6a47x/?view_only=b9fd80cc7cff45a1bc64bd81aad574a8.
