# Author Correction: Weight Bearing Activities change the Pivot Position after Total Knee Arthroplasty

**DOI:** 10.1038/s41598-020-57831-z

**Published:** 2020-01-15

**Authors:** Philippe Moewis, Hagen Hommel, Adam Trepczynski, Leonie Krahl, Philipp von Roth, Georg N. Duda

**Affiliations:** 1 Julius Wolff Institute, Charité - Universitätsmedizin Berlin, Berlin, Germany; 2 Krankenhaus Märkisch-Oderland GmBH, Wriezen, Germany; 3 Medizinischen Hochschule Brandenburg Theodor Fontane, Neuruppin, Germany; 4 Sporthopaedicum Regensburg, Regensburg, Germany

Correction to: *Scientific Reports* 10.1038/s41598-019-45694-y, published online 24 June 2019

The Acknowledgements section in this Article is incomplete.

“This work was supported by Implantcast GmbH, the European Regional Development Fund (EFRE, 16409608, OrthoLoadLab), the Federal Ministry of Education and Research (BMBF, OVERLOAD-PrevOP, 01EC1408A), the German Research Foundation (DFG, TR 1657/1-1) and the OrthoLoad Club. We also acknowledge support from the German Research Foundation (DFG) and the Open Access Publication Funds of Charité – Universitätsmedizin Berlin.”

should read:

“This work was supported by Implantcast GmbH, the European Regional Development Fund (EFRE, 16409608, OrthoLoadLab), the Federal Ministry of Education and Research (BMBF, OVERLOAD-PrevOP, 01EC1408A), the German Research Foundation (DFG, TR 1657/1-1) and the OrthoLoad Club. We also acknowledge support from the German Research Foundation (DFG) and the Open Access Publication Funds of Charité – Universitätsmedizin Berlin. Additionally, we would like to thank Dr. Sabine Schmitt and her team at the Krankenhaus Martha-Maria Halle Dölau for their support during patient selection, recruitment and general organization of the study.”

