# Supplementary material for: Weight Bearing Activities change the Pivot Position after Total Knee Arthroplasty
Source: Sci Rep. 2019 Jun 24;9:9148. doi: 10.1038/s41598-019-45694-y (PMC6591446; doi:10.1038/s41598-019-45694-y)
Supplement: Supplementary file 1 — Supplementary Info [file 41598_2019_45694_MOESM1_ESM.pdf]

Weight Bearing Activities change the Pivot Position after Total Knee Arthroplasty

Philippe Moewis, Hagen Hommel, Adam Trepczynski, Leonie Krahl, Philipp von Roth, Georg N. Duda

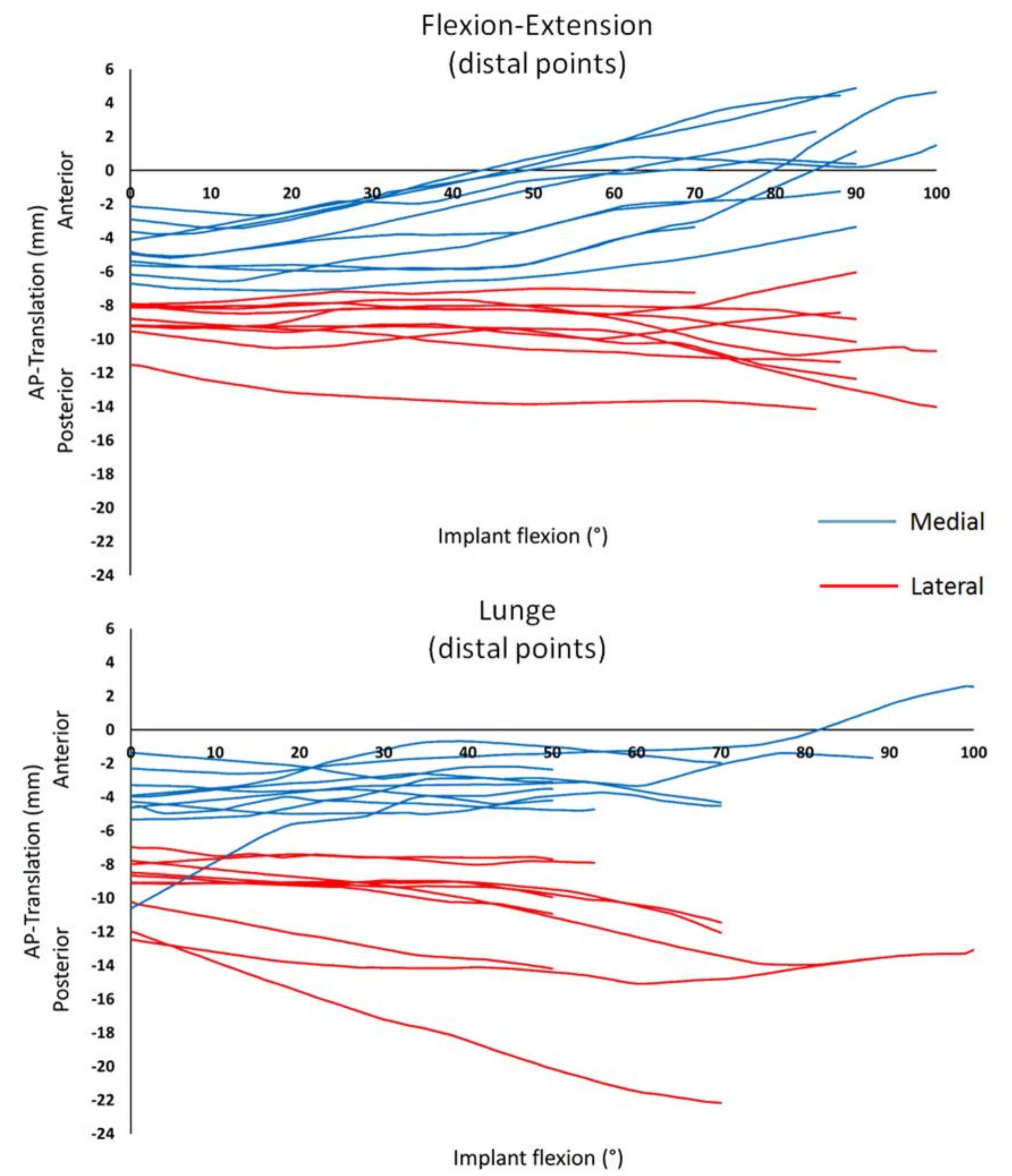

Appendix Figure 1: Medial and lateral distal points anterior-posterior translation during unloaded flexion-extension and weight-bearing lunge.

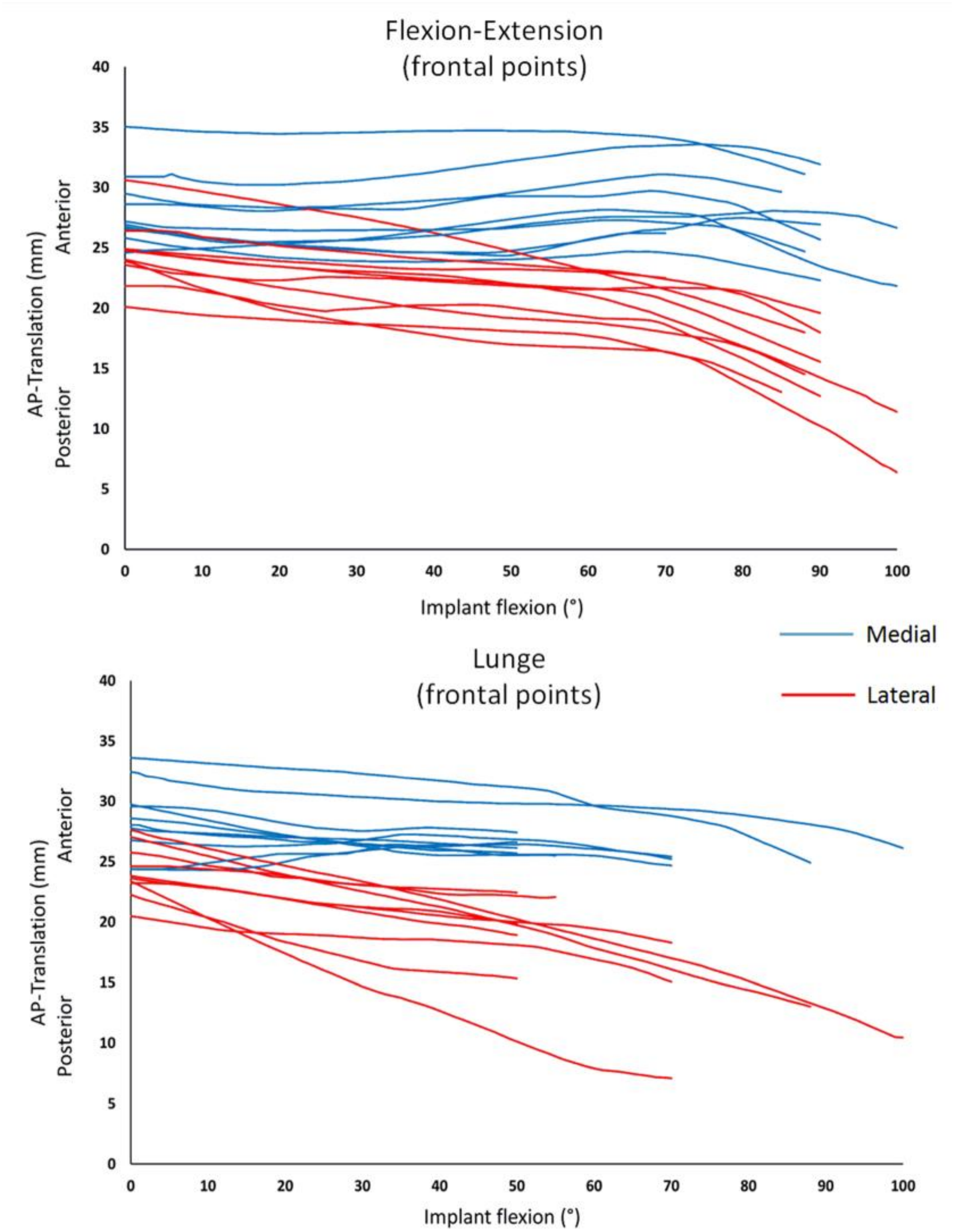

Appendix Figure 2: Medial and lateral frontal points anterior-posterior translation during unloaded flexion-extension and weight-bearing lunge.

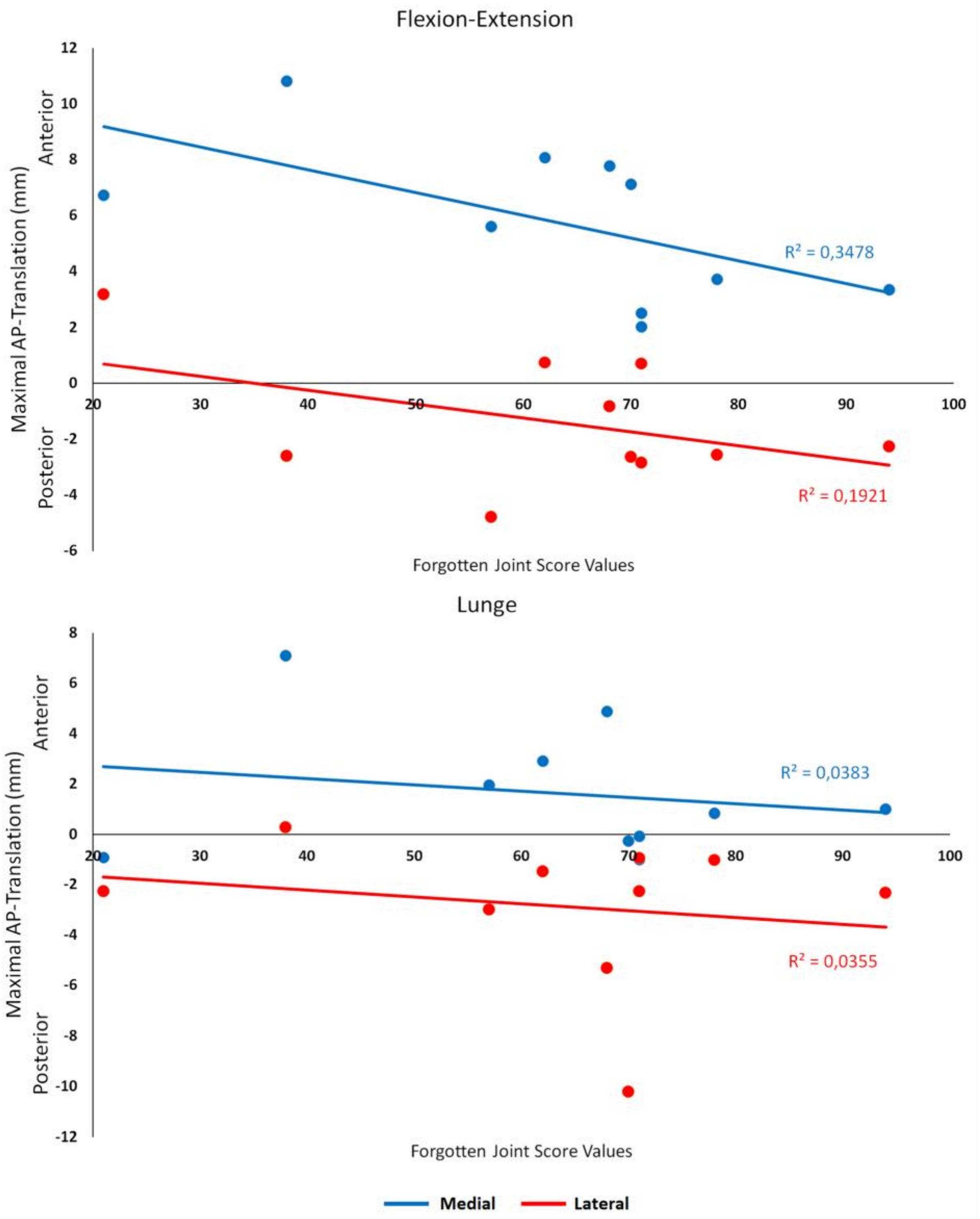

Appendix Figure 3: Correlation analysis between the FJS values and the AP translation at maximal achieved knee joint flexion for both the medial and lateral compartments.
